# Precision immunopharmacology in peri-implantitis management: from molecular mechanisms to advanced therapeutic strategies

**DOI:** 10.3389/fimmu.2026.1748582

**Published:** 2026-05-26

**Authors:** Yong Chen, Qing Yuan, MingWang Cui, ZhuLing Guo

**Affiliations:** 1 School of Dentistry, Hainan Medical University, Haikou, China; 2 Department of Health Management Center, The First Affiliated Hospital of Hainan Medical University, Haikou, China

**Keywords:** biomarkers, drug delivery, immunopharmacology, osteoimmunology, peri-implantitis, precision medicine

## Abstract

**Objectives:**

To review the evolving shift in peri-implantitis research from traditional mechanical debridement toward host-modulatory and immunopharmacological concepts, focusing on molecular pathogenesis, candidate therapeutic targets, and advanced drug delivery systems requiring further validation.

**Methods:**

A comprehensive literature review was conducted to synthesize current evidence on the osteoimmunology of peri-implantitis, including findings from single-cell RNA sequencing, studies on host-modulation therapies, and developments in nanotechnology-based delivery platforms.

**Results:**

Peri-implantitis is increasingly recognized as an immunologically complex condition, with descriptive single-cell studies suggesting cell-interaction patterns such as the CXCL13+ fibroblast-CXCR2+ neutrophil axis. Candidate therapeutic strategies include targeted anti-cytokine biologics, specialized pro-resolving mediators (SPMs), and macrophage-directed approaches, but most remain preclinical or early translational. Advanced delivery systems such as stimuli-responsive hydrogels and exosome-based platforms may support localized treatment, while machine-learning models show promise for risk stratification but require prospective clinical validation.

**Conclusions:**

Emerging immunopharmacological strategies, together with biomarker diagnostics and artificial intelligence, may help move peri-implantitis management toward more proactive and personalized care, provided that their mechanistic relevance and clinical utility are validated in rigorous studies.

## 1 Introduction

Dental implant therapy has emerged as the gold standard for tooth replacement, with over 2 million implants placed annually in the United States alone (1). However, the increasing prevalence of peri-implantitis, affecting 8.9-45% of patients with dental implants, poses a significant challenge to long-term treatment success (2, 3). Traditional therapeutic approaches, primarily focused on mechanical debridement and antimicrobial therapy, have demonstrated limited efficacy with recurrence rates reaching 65% (4, 5). This therapeutic inadequacy has prompted a fundamental reassessment of peri-implantitis pathogenesis and treatment strategies.

The emergence of precision medicine in dentistry reflects broader healthcare trends toward personalized, biomarker-guided interventions (6). Recent technological advances, including single-cell RNA sequencing, artificial intelligence, and nanotechnology-based drug delivery systems, have expanded our understanding of peri-implant immune responses (7). These developments suggest a potential shift from empirical treatment toward more mechanism-informed immunopharmacological interventions, although translation into routine clinical practice remains incomplete (8).

The economic burden of peri-implantitis extends beyond individual patient outcomes, with projected healthcare costs reaching $3.2 billion by 2033. This substantial financial impact, combined with the psychological and functional consequences for patients, underscores the urgent need for more effective therapeutic strategies. Current evidence suggests that the future of peri-implantitis management lies in understanding and modulating the complex interplay between microbial biofilms, host immune responses, and biomaterial interactions (9, 10).

This comprehensive review examines the molecular foundations of peri-implantitis, evaluates emerging immunopharmacological interventions, and explores advanced drug delivery technologies with potential for localized therapeutic targeting. We synthesize current evidence while distinguishing validated clinical findings from plausible mechanistic hypotheses and future translational possibilities involving molecular diagnostics, artificial intelligence-guided risk stratification, and targeted immunomodulation.

## 2 Molecular foundations of peri-implantitis: beyond traditional paradigms

### 2.1 Redefining osseointegration as an osteoimmune phenomenon

The traditional concept of osseointegration as a passive healing process has been fundamentally challenged by emerging evidence from osteoimmunology research (11, 12). Contemporary understanding positions osseointegration as an active, dynamic process orchestrated by immune cells, particularly macrophages and their interactions with osteoprogenitor cells (13). This paradigm shift reframes successful implant integration as the establishment of a “foreign body equilibrium” (FBE), wherein the host immune system actively isolates the implant through controlled bone formation while maintaining tissue homeostasis (14).

The critical role of macrophage polarization in determining osseointegration outcomes has been extensively documented. Successful integration requires a timely transition from pro-inflammatory M1 macrophages to regenerative M2 phenotypes (15, 16). This transition is mediated by complex signaling networks involving Toll-like receptors, cytokine cascades, and mechanotransduction pathways (17). Disruption of this delicate balance, either through excessive inflammatory stimuli or impaired resolution mechanisms, can lead to persistent inflammation and eventual peri-implantitis development (18).

Recent studies have identified osteomacs, specialized bone-resident macrophages, as key regulators of bone homeostasis around implants (15). These cells, distinct from circulating monocyte-derived macrophages, maintain intimate contact with osteoblasts and osteoclasts, serving as local coordinators of bone remodeling responses. Understanding osteomac biology opens new therapeutic avenues for modulating peri-implant bone metabolism through targeted immunomodulation.

### 2.2 Single-cell transcriptomics reveals disease-specific signatures

Single-cell RNA sequencing (scRNA-seq) studies have provided important descriptive insights into peri-implantitis pathogenesis and suggest that peri-implant lesions may exhibit pathological features that differ from periodontitis and healthy tissues (19). Li and colleagues’ 2024 study reported distinct immune microenvironment characteristics in peri-implantitis; however, these findings should be interpreted as hypothesis-generating until supported by causal and interventional validation.

Recent scRNA-seq analyses of periodontal and peri-implant tissues have identified a CXCL13+ fibroblast subpopulation within the peri-implant microenvironment (19). Emerging evidence within oral inflammatory disease models suggests that these fibroblasts may interact with CXCR2+ neutrophils via local chemokine networks, potentially amplifying fibroblast activation and osteoclastogenic signaling (20). However, it remains unclear whether this specific axis is a primary driver of bone loss, a secondary feature of chronic inflammation, or a context-dependent interaction. Therefore, the CXCL13+ fibroblast-CXCR2+ neutrophil axis should currently be viewed as a descriptive and hypothesis-generating mechanism rather than a validated peri-implantitis-specific therapeutic target. Beyond this axis, dynamic interactions among osteal macrophages, osteoclasts, and osteoblast-lineage cells contribute to the balance of bone remodeling.

**Figure 1:**
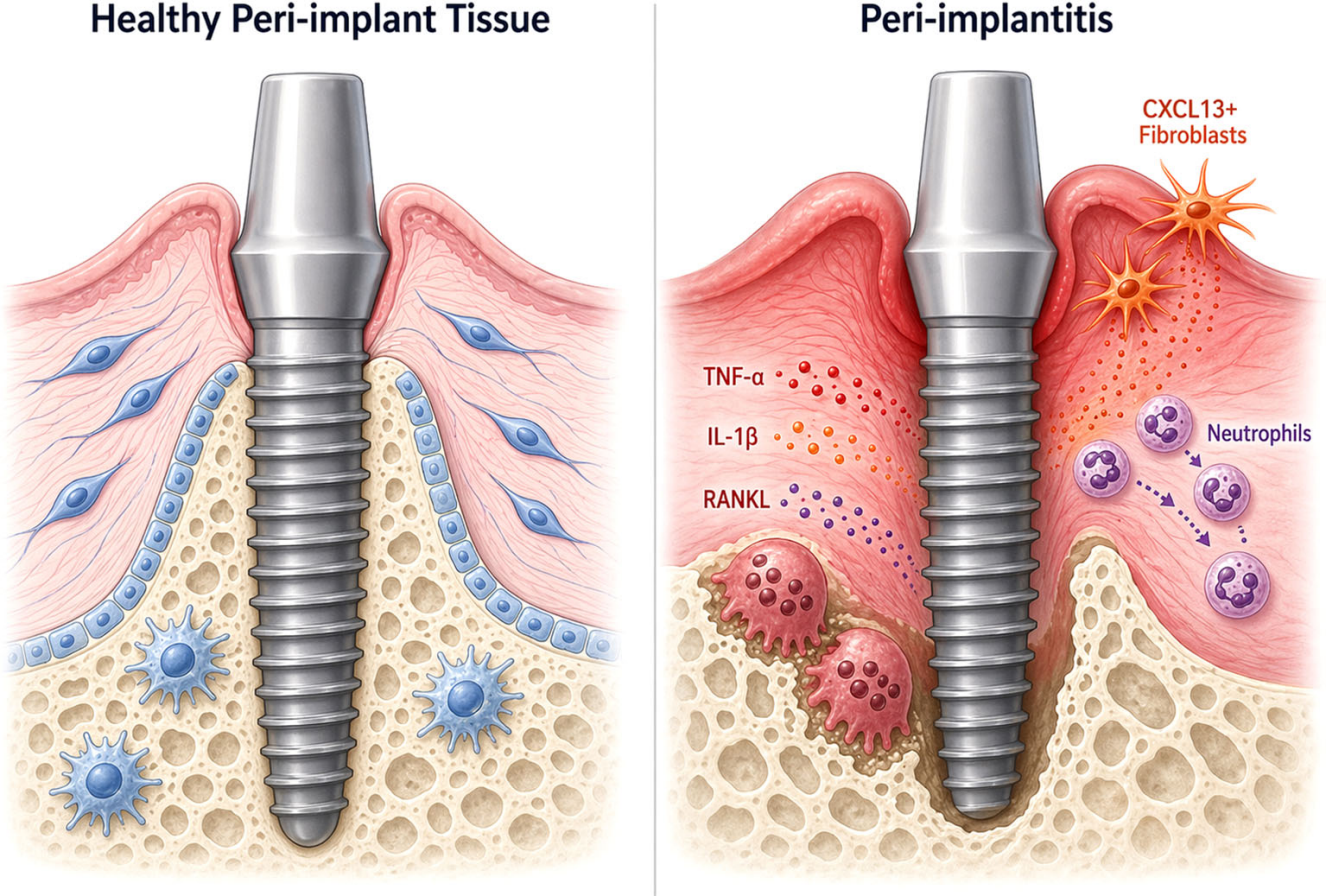
Schematic illustration of the cellular landscape and candidate signaling axes in the peri-implantitis microenvironment. This figure provides a comparative overview of the cellular composition in healthy peri-implant tissues versus peri-implantitis lesions. The left panel (Homeostasis) depicts the tissue architecture under stable conditions, characterized by quiescent normal fibroblasts, bone-lining osteoblasts, and bone-resident macrophages (osteomacs) that maintain local tissue homeostasis. The right panel (Peri-implantitis) illustrates the pathological transformation of the microenvironment, including the proposed CXCL13+ fibroblast-CXCR2+ neutrophil interaction axis identified through single-cell transcriptomic analysis. Current evidence suggests that this interaction may contribute to neutrophil recruitment and inflammatory amplification, but its causal role and therapeutic specificity remain to be validated. This process is accompanied by elevated levels of pro-inflammatory cytokines (e.g., TNF-α, IL-1β) and the osteoclastogenic factor RANKL, contributing to bone resorption and peri-implant tissue destruction.

Machine learning analysis of scRNA-seq data has identified immune signature patterns that correlate with disease severity and exploratory prognostic categories (21). High-risk patient groups in these datasets demonstrate elevated CD4+ T cell infiltration, increased IL-1β and MMP-9 levels in peri-implant crevicular fluid, and microbiome enrichment by Fusobacterium nucleatum and Prevotella intermedia (22). These molecular signatures provide a rationale for future precision diagnostic studies and personalized treatment hypotheses, but they do not yet constitute clinically validated decision rules (**Figure 1**).

### 2.3 Dysregulated immune networks: from innate to adaptive responses

Peri-implantitis pathogenesis involves complex dysregulation of both innate and adaptive immune responses (23, 24). The innate immune system, primarily through pattern recognition receptors (PRRs), initiates inflammatory cascades upon detection of bacterial components and titanium particles (25). Toll-like receptor 2 and 4 activation leads to MyD88-dependent signaling, promoting production of pro-inflammatory cytokines including TNF-α, IL-1β, and IL-6 (26).

The adaptive immune response in peri-implantitis demonstrates characteristic T helper cell subset imbalances (27). Th1 and Th17 cell populations predominate, while regulatory T cells (Tregs) are functionally impaired or numerically depleted (28). This imbalance perpetuates inflammatory responses and prevents effective resolution. IL-17 production by Th17 cells directly promotes osteoclastogenesis and neutrophil recruitment, creating positive feedback loops that sustain tissue destruction (29, 30).

B cell responses in peri-implantitis remain incompletely understood, though emerging evidence suggests important roles in antibody production against titanium-associated antigens and cytokine secretion (31). The presence of ectopic lymphoid structures in severe peri-implantitis lesions indicates organized adaptive immune responses that may contribute to disease persistence (32).

#### 2.3.1 Immunometabolic reprogramming in peri-implantitis

The transition of immune cells into pathogenic phenotypes in the peri-implant pocket is fundamentally driven by immunometabolic reprogramming (33). Pro-inflammatory M1-like macrophages and activated neutrophils increasingly rely on aerobic glycolysis (the Warburg effect) rather than oxidative phosphorylation (34). This metabolic shift, regulated by HIF-1α and mTOR signaling, ensures rapid ATP production for cytokine synthesis and ROS generation. Consequently, the accumulation of lactate from glycolysis acidifies the local peri-implant microenvironment, further activating matrix metalloproteinases (MMPs) and accelerating bone resorption.

#### 2.3.2 Epigenetic regulation of the osteoimmune landscape

Sustained pro-inflammatory states in peri-implantitis are additionally anchored by epigenetic modifications (35). Chronic exposure to biofilm-derived LPS and titanium dissolution products induces long-lasting alterations in DNA methylation and histone acetylation within tissue-resident cells (36). These epigenetic shifts silence anti-inflammatory pathways while keeping pro-inflammatory gene promoters (e.g., TNF-α, IL-1β) highly accessible. Understanding these underlying metabolic and epigenetic landscapes is crucial, as they explain why simple mechanical debridement often fails to reverse the established pathological cellular phenotypes.

### 2.4 Molecular mechanisms of bone destruction

The RANKL/RANK/OPG axis represents the central regulatory pathway governing osteoclast differentiation and bone resorption in peri-implantitis (37, 38). Meta-analyses consistently demonstrate significantly elevated RANKL levels and increased RANKL/OPG ratios in diseased sites compared to healthy tissues (39). This pathway dysregulation directly promotes osteoclastogenesis through NF-κB activation and TRAF6 signaling (40).

Beyond classical RANKL-dependent mechanisms, inflammatory cytokines can directly stimulate osteoclast formation and activation (41). TNF-α and IL-6 bypass RANKL requirements, enabling continued bone resorption even in the presence of RANKL inhibitors (42). This “inflammatory osteolysis” phenomenon explains the limited efficacy of single-target therapeutic approaches and supports multi-modal intervention strategies (43).

Recent discoveries have identified novel signaling pathways contributing to peri-implant bone loss. The LOX-1/ERK1/2 pathway mediates P. gingivalis-induced RANKL expression through TLR2 and ERK1/2 signaling while regulating MMP9 expression (44). This pathway represents an innovative therapeutic target linking oxidative stress responses to matrix degradation and inflammatory perpetuation (45).

## 3 Current therapeutic landscape: limitations and opportunities

### 3.1 Conventional approaches: efficacy and limitations

Traditional peri-implantitis treatment relies primarily on mechanical debridement, often combined with antimicrobial therapy (46, 47). However, multiple systematic reviews and meta-analyses have demonstrated the limited efficacy of these approaches, particularly for established peri-implantitis (48, 49). Cochrane reviews consistently report no significant additional benefits from systemic or local antibiotic therapy compared to mechanical treatment alone (50, 51).

The fundamental limitation of antimicrobial-focused strategies lies in their failure to address the underlying immune dysregulation driving disease progression (52). While bacterial biofilms certainly contribute to disease initiation and perpetuation, the host inflammatory response represents the primary determinant of tissue destruction (53). This recognition has prompted increased interest in host-modulation therapies targeting immune and inflammatory pathways (54).

Non-steroidal anti-inflammatory drugs (NSAIDs) present a therapeutic paradox in peri-implantitis management (55). While these agents provide potent anti-inflammatory effects, their inhibition of cyclooxygenase-2 interferes with essential bone healing processes (56). Animal studies and retrospective human data suggest that NSAID use may impair osseointegration and increase implant failure risk (57, 58). This contradiction highlights the need for more sophisticated anti-inflammatory strategies that preserve beneficial healing responses while controlling pathological inflammation.

### 3.2 Emerging host modulation strategies

Recognition of peri-implantitis as an immune-mediated disease has led to exploration of various host modulation therapies (59, 60). These approaches target specific inflammatory mediators or immune cell populations rather than broadly suppressing immune function (61). Several promising strategies have emerged from this research direction.

Sub-antimicrobial dose doxycycline represents one of the first clinically successful host modulation therapies (62). Beyond its antimicrobial effects, doxycycline inhibits matrix metalloproteinases (MMPs) that contribute to tissue destruction (63). Clinical trials have demonstrated modest improvements in clinical parameters when doxycycline is used as an adjunct to mechanical therapy (64).

Bisphosphonates and other anti-resorptive agents have shown promise in preclinical models through their ability to inhibit osteoclast function (65). However, clinical application is limited by concerns regarding medication-related osteonecrosis of the jaw (MRONJ) (66). Local delivery strategies may mitigate systemic risks while maintaining therapeutic efficacy (67).

Statins have emerged as promising host modulation agents due to their pleiotropic effects beyond cholesterol-lowering (68). These drugs demonstrate anti-inflammatory properties, promote osteoblast differentiation, and inhibit osteoclast formation (69). Local statin delivery has shown encouraging results in periodontal regeneration studies (70).

## 4 Precision immunopharmacological interventions

### 4.1 Targeted anti-cytokine therapies

The identification of cytokine networks associated with peri-implantitis pathogenesis has motivated investigation of targeted biological therapies. TNF-α antagonists, including adalimumab, infliximab, and etanercept, provide mechanistic specificity through TNF-α blockade and may attenuate downstream inflammatory cascade activation in relevant inflammatory models (71).

Preclinical studies using TNF-α inhibitors in experimental periodontitis models have shown significant reductions in inflammation, neutrophil infiltration, and bone loss (72). Clinical evidence from rheumatoid arthritis patients receiving anti-TNF therapy demonstrates improved periodontal status and reduced gingival crevicular fluid TNF-α levels (73). These findings support the therapeutic potential of TNF-α blockade in peri-implantitis management.

IL-17 pathway inhibitors represent another promising therapeutic avenue (74). Secukinumab and other IL-17A antagonists address the elevated IL-17 levels consistently observed in peri-implant crevicular fluid (75). The association between IL-17A genetic polymorphisms and peri-implantitis susceptibility further supports targeting this pathway (76).

JAK inhibitors offer unique advantages through their ability to simultaneously target multiple cytokine pathways via JAK-STAT signaling inhibition (77). Tofacitinib, baricitinib, and other JAK inhibitors affect IL-6, IL-23, and IFN-γ pathways through oral administration, potentially simplifying treatment protocols while improving patient compliance (78).

### 4.2 Specialized pro-resolving mediators: paradigm shift to resolution pharmacology

Specialized pro-resolving mediators (SPMs) represent an emerging approach to inflammation management (79, 80). Unlike traditional anti-inflammatory drugs that primarily suppress inflammatory responses, SPMs can promote inflammation resolution through endogenous pathways in experimental models (81). Resolvins, protectins, and maresins derived from omega-3 fatty acids therefore represent promising, but still translational, therapeutic candidates (82).

It is critical to distinguish SPMs from targeted anti-cytokine biologics (discussed in Section 4.1). Anti-cytokine therapies aim to suppress or neutralize specific inflammatory drivers, effectively “braking” the inflammatory cascade but potentially leaving the tissue in a vulnerable, non-healing state. In contrast, SPMs are actively pro-resolving; they can accelerate neutrophil apoptosis, enhance macrophage efferocytosis, and support tissue repair in experimental contexts (83). A plausible future paradigm may involve sequential administration: initial application of anti-cytokine biologics to dampen severe inflammation, followed by localized delivery of SPMs to encourage resolution and bone regeneration. This concept remains speculative for peri-implantitis and requires dedicated preclinical and clinical testing.

RvD1, RvD2, and RvE1 have been reported to inhibit neutrophil infiltration, enhance macrophage-mediated clearance of apoptotic cells through efferocytosis, and promote tissue repair in relevant models (84, 85). Available evidence suggests that RvD2 may limit tissue damage by controlling neutrophil influx and modulating osteoclast activity, but direct peri-implantitis clinical evidence remains limited (86). Importantly, SPMs may avoid some adverse effects associated with conventional anti-inflammatory drugs, although safety and efficacy require indication-specific testing (87).

**Figure 2:**
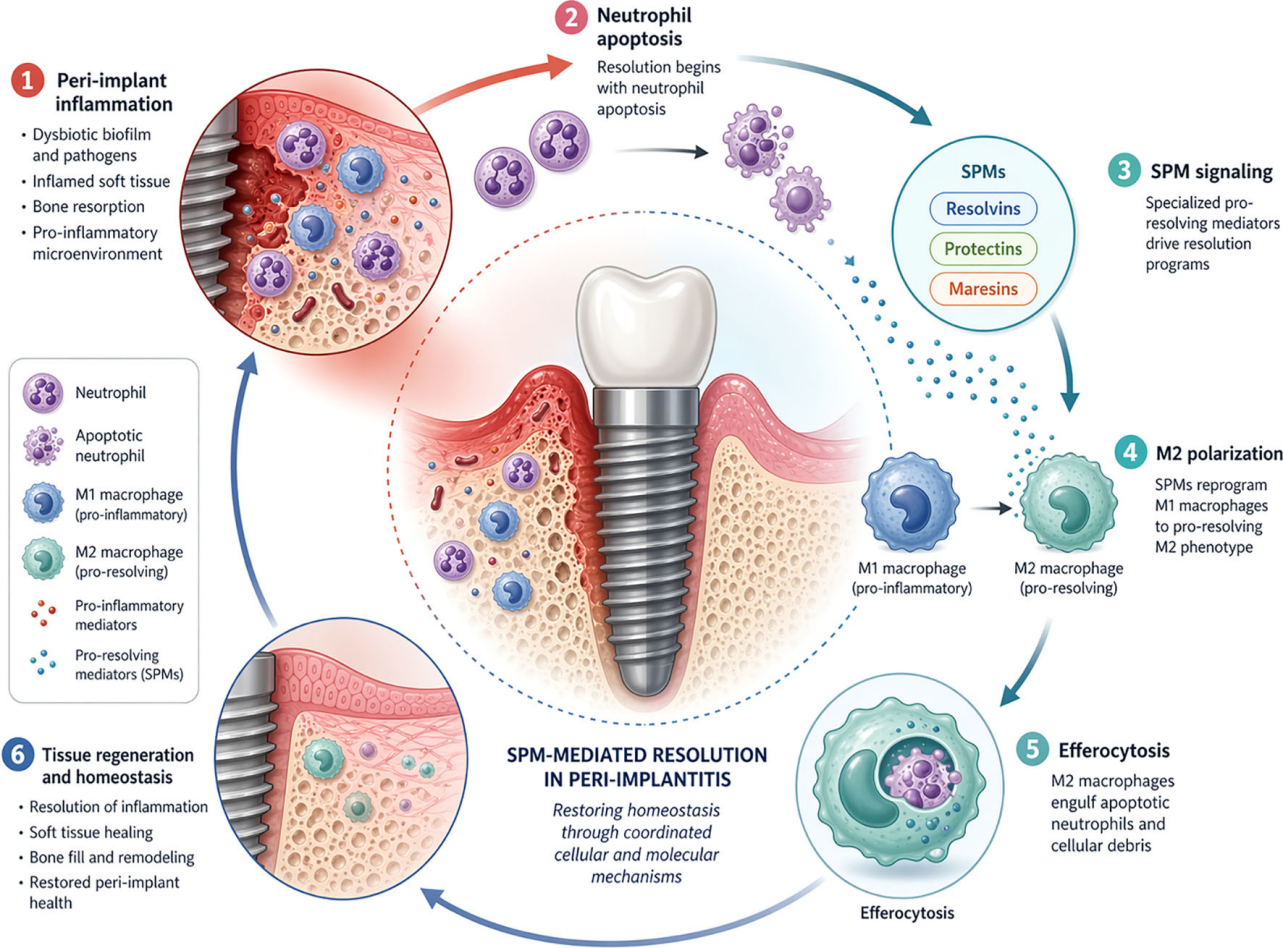
Proposed mechanisms of specialized pro-resolving mediators (SPMs) in the resolution of peri-implant inflammation. This schematic illustrates a proposed inflammation-resolution cycle mediated by SPMs in the peri-implant environment. The process includes inhibition of neutrophil infiltration and promotion of neutrophil apoptosis. Centrally, the figure depicts a possible phenotypic switch of macrophages from a pro-inflammatory M1 state to a pro-resolving M2 phenotype, characterized by their distinct mononuclear morphology. These M2 macrophages may facilitate efferocytosis, the clearance of apoptotic neutrophils and cellular debris, which is a prerequisite for tissue repair. Unlike conventional anti-inflammatory approaches that primarily suppress inflammation, this SPM-guided pathway is proposed to guide the microenvironment toward tissue regeneration and restoration of homeostasis.

A phase I clinical trial using stable lipoxin A4 analog (BLXA4) as a mouthrinse for gingivitis treatment demonstrated safety and preliminary efficacy (88). The treatment reduced local gingival inflammation and altered serum lipid mediator profiles, increasing SPM levels while decreasing pro-inflammatory eicosanoids. These findings support further investigation of resolution pharmacology approaches, but they should not be directly extrapolated to peri-implantitis without disease-specific trials (**Figure 2**).

### 4.3 Macrophage-targeted therapeutics

Macrophage polarization represents a critical therapeutic target given these cells’ central role in determining disease outcomes (89, 90). Strategies to eliminate pro-inflammatory M1 macrophages or reprogram them toward anti-inflammatory M2 phenotypes show significant promise (91).

Clodronate-loaded liposomes can deplete macrophages through phagocytosis-induced apoptosis (92). Preclinical studies suggest that macrophage depletion may reduce peri-implant bone loss and decrease TNF-α levels in experimental settings (93). However, non-selective macrophage elimination may impair beneficial immune functions, necessitating more sophisticated targeting approaches before clinical translation (94).

M2 polarization strategies using IL-4, IL-13, or other anti-inflammatory mediators represent more nuanced approaches (95). Nanoparticle delivery systems are being investigated to enrich polarization signals within inflammatory macrophage populations, with the goal of promoting phenotypic switching while limiting off-target effects (96). Natural compounds such as resveratrol have shown efficacy in promoting M1-to-M2 transition in periodontal disease models (97).

Given the focus on macrophage-targeted therapeutics, it is essential to consider the phenomenon of “trained immunity” (innate immune memory) (98). Repeated exposure to peri-implant pathogens and titanium particles may induce epigenetic and metabolic reprogramming in resident macrophages, rendering them hyper-responsive to subsequent stimuli (99). Whether current immunopharmacological agents can reverse this altered landscape remains uncertain. Future precision therapeutics may need to explore epigenetic modifiers (e.g., selected HDAC inhibitors) or metabolic modulators as candidate approaches to attenuate pathogenic innate immune memory and restore more physiological immune surveillance.

### 4.4 Regenerative immunomodulation

The integration of regenerative medicine with immunomodulation represents a potential therapeutic strategy addressing both inflammatory control and tissue reconstruction (100, 101). Platelet-derived growth factor (PDGF) applications have shown improved bone regeneration when combined with bone grafts in selected contexts (102). Recent case reports of rhPDGF combined with allografts describe favorable treatment of challenging periodontal intrabony defects (103), suggesting a rationale for carefully designed peri-implant studies.

Stem cell therapies utilizing periodontal ligament stem cells combined with BMP-2 expression have shown defect regeneration and enhanced re-osseointegration in experimental models (104). Mesenchymal stem cells demonstrate both immunomodulatory and regenerative properties, making them plausible candidates for combination therapies that still require peri-implantitis-specific validation (105).

The combination of SPMs with regenerative protocols represents cutting-edge tissue engineering that actively promotes inflammation resolution while enhancing repair processes (106). This approach moves beyond simple anti-inflammatory suppression toward active promotion of healing responses (107).

## 5 Advanced drug delivery technologies

### 5.1 Smart hydrogel systems

Injectable hydrogels have emerged as attractive drug carriers for peri-implant applications because they can conform to irregular defect geometries while providing sustained drug release (108, 109). Smart hydrogels incorporate stimuli-responsive properties that may allow controlled drug release in response to disease-associated environmental changes (110).

Temperature-sensitive PEG-PLGA-PEG triblock copolymers undergo sol-gel transitions at physiological temperatures, enabling injectable liquid delivery that forms solid drug depots in situ (111). pH-sensitive chitosan-based hydrogels exploit the acidic microenvironment characteristic of peri-implantitis sites (112). MMP-responsive hydrogels incorporate cleavable peptide sequences that respond directly to elevated MMP-8 levels, a key inflammatory biomarker (113).

Recent innovations include ROS-responsive hydrogels utilizing boronate ester bonds that remain stable under normal conditions but cleave in high ROS environments (114). These systems are designed to support conditional, site-enriched drug release in response to inflammatory cues. Co-delivery platforms capable of releasing multiple therapeutic agents may produce synergistic effects through simultaneous antimicrobial, anti-inflammatory, and regenerative interventions, although peri-implantitis-specific evidence remains largely preclinical (115).

### 5.2 Metal-organic frameworks for enhanced drug loading

Metal-organic frameworks (MOFs) represent a promising technology for drug loading and protection (116, 117). ZIF-8, UiO-66, and MIL-88A systems have achieved up to 50% weight-to-weight drug loading in reported experimental settings while protecting labile therapeutic agents from degradation (118). MOF integration with hydrogels may provide sustained release capabilities relevant to chronic inflammatory conditions, but peri-implantitis-specific performance remains to be established (119).

Recent applications of simvastatin@ZIF-8 systems demonstrate the feasibility of anti-inflammatory therapy delivery with controlled release kinetics (120). The modular nature of MOF systems may allow customization for specific therapeutic agents and release profiles (121). However, long-term biocompatibility, biodegradation patterns, and peri-implant tissue responses require further investigation before clinical translation.

### 5.3 Exosome-based targeted delivery

Exosomes are being investigated as biological drug delivery systems (122, 123). These 30–150 nanometer extracellular vesicles may offer natural tropism and relatively low immunogenicity profiles in selected contexts (124). Loading efficiencies of 20-26% have been reported through electroporation and sonication methods, although reproducibility and clinical scalability remain important challenges (125).

**Figure 3:**
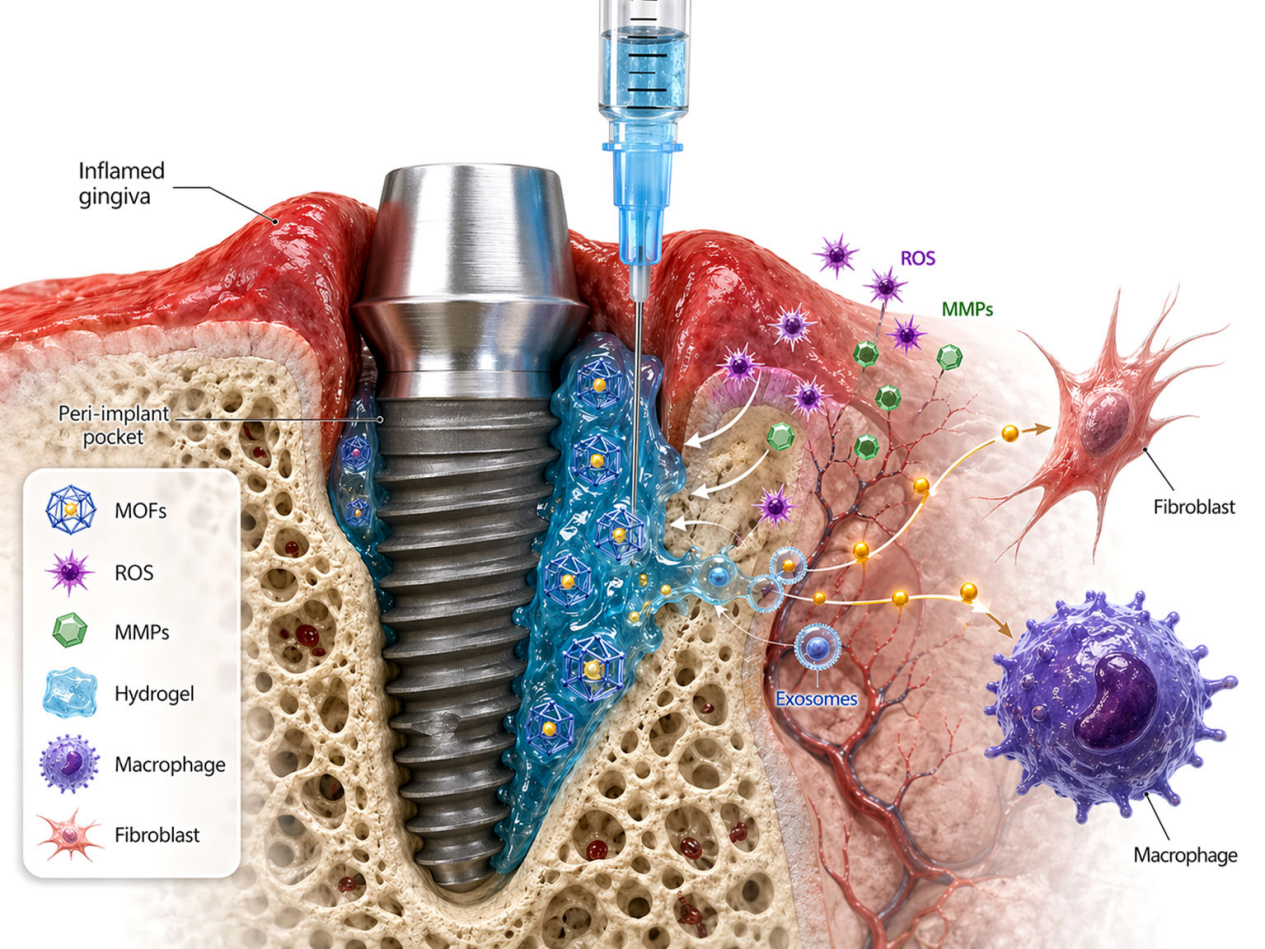
Advanced candidate drug delivery platforms for localized peri-implantitis management. This schematic illustration depicts the localized administration and proposed mechanistic pathways of a stimuli-responsive hydrogel system. The therapeutic hydrogel is delivered via a minimally invasive vertical injection into the inflamed peri-implant pocket, where in situ sol-gel transition may help retain the therapeutic depot. Within the hydrogel matrix, metal-organic frameworks (MOFs) and exosome-inspired vesicles encapsulate therapeutic agents, potentially improving drug loading and protection against premature degradation. The system is designed to exhibit conditional release kinetics triggered by microenvironmental hallmarks of peri-implantitis; reactive oxygen species (ROS) and matrix metalloproteinases (MMPs) may cleave responsive bonds of the carrier. Released therapeutic molecules are intended to interact with local effector cells, including resident macrophages and fibroblasts, to modulate the osteoimmune microenvironment and support tissue regeneration. These mechanisms remain largely preclinical and require peri-implantitis-specific validation.

Mesenchymal stem cell-derived exosomes demonstrate particular promise for regenerative applications. Recent studies in dental and craniofacial regeneration report improved healing in bone-defect models, but these findings cannot yet be directly generalized to peri-implantitis defects. Exosome surface modification may enhance enrichment in selected cell populations and improve therapeutic specificity in future studies (**Figure 3**).

### 5.4 Three-dimensional printed scaffolds

Additive manufacturing technologies may support patient-specific therapeutic delivery through CT/MRI-guided custom scaffold design (126, 127). Three-dimensional printed drug-eluting scaffolds can provide programmable drug release from multiple layers with spatial and temporal control in experimental systems (128). PLGA scaffolds incorporating 5-fluorouracil demonstrate aperture size-dependent release kinetics (129).

Biodegradable PLA/β-TCP composites for craniofacial reconstruction show promise in preclinical models (130). However, clinical translation faces challenges in regulatory approval for patient-specific devices and quality control standardization (131). Multi-material printing may allow incorporation of different therapeutic agents in distinct scaffold regions, but this remains an engineering concept for peri-implantitis applications (132).

### 5.5 Microneedle patches for minimally invasive delivery

Microneedle patches represent an innovative strategy for overcoming barriers to local drug delivery (133, 134). These systems are designed to penetrate superficial tissues with limited discomfort while reducing systemic exposure and salivary washout (135). Dissolving microneedles may allow controlled release through biodegradable polymer matrices, but their use in peri-implant pockets requires specific safety and retention studies (136).

Recent innovations include modular, multi-stage release systems with burst release of antibiotics from dissolvable backing films and sustained release of immunomodulatory factors from biodegradable needle bodies (137). Animal studies in related delivery contexts demonstrate inflammation suppression, enhanced M2 macrophage formation, and tissue healing. The potential for patient self-administration is attractive for treatment compliance, but its feasibility and safety in peri-implant pockets remain to be established rather than inferred from generalized transdermal delivery models (138).

## 6 Artificial intelligence and precision diagnostics

### 6.1 Machine learning applications in risk stratification

Artificial intelligence applications have shown promising performance in image-based peri-implantitis detection and implant-related measurements. Deep learning models utilizing YOLOv7 implementations have reported 97.10% F1 scores for peri-implantitis detection in periapical radiographs, and U-Net architectures have achieved high accuracy for implant segmentation and automated measurement in selected datasets (139). These results support further validation but should not be equated with prospective clinical prognostic accuracy.

Beyond image-based detection, transcriptomic deconvolution and machine-learning models developed in other biomedical contexts may provide methodological references for future peri-implantitis risk stratification (140). However, their applicability to peri-implantitis prognosis or treatment-response prediction remains unproven. Prospective peri-implantitis-specific datasets, external validation, and clinically meaningful outcome endpoints are required before these approaches can be used for clinical decision support.

### 6.2 Biomarker discovery and point-of-care diagnostics

The discovery of candidate biomarker genes, including ACTA2, FAP, and PDGFRβ, may help distinguish peri-implantitis from periodontitis at the molecular level. At present, these markers should be considered exploratory signatures that require independent validation before they can guide diagnostic differentiation or targeted therapeutic selection (141).

FDA-approved point-of-care diagnostic systems, including PerioSafe PRO DRS and ImplantSafe DR, represent first-generation chair-side biomarker tools (142). These systems utilize aMMP-8 detection for rapid biomarker assessment and may complement, rather than replace, clinical and radiographic evaluation. Integration with electronic health records could facilitate longitudinal monitoring and future treatment optimization, provided that clinical utility is demonstrated (143).

### 6.3 Personalized treatment algorithms

The Implant Disease Risk Assessment tool has provided clinically derived risk stratification, with reported 69% accuracy in identifying patients who developed peri-implantitis in the evaluated cohort (144). Low-risk patients demonstrated 0% incidence, while high-risk categories showed 27.1% disease development. Bleeding on probing emerged as the most significant predictive parameter, but broader external validation is needed before universal clinical application (145).

**Figure 4:**
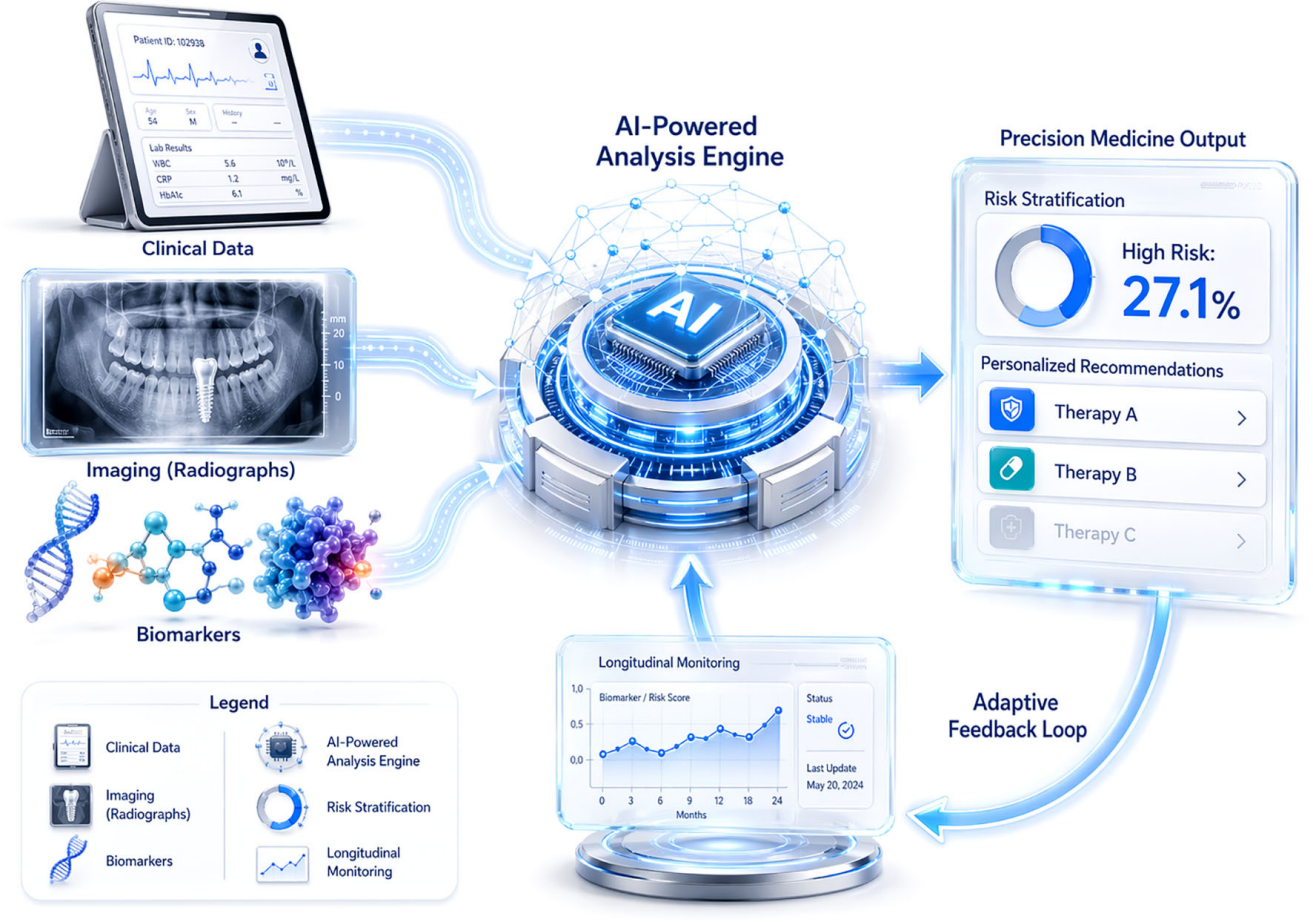
Conceptual workflow for artificial intelligence-supported diagnostics and treatment selection. This figure presents a proposed AI-assisted analysis engine for integrating multi-modal data in peri-implant care. The engine ingests diverse input data, including digitized clinical records, radiographic imaging for automated bone loss measurement, and molecular biomarker profiles (e.g., aMMP-8 and candidate gene signatures). Through deep learning and predictive analytics, the system may support individual risk stratification and identification of higher-risk patient cohorts. A decision-support interface could suggest candidate therapeutic options based on the patient’s immune profile. The workflow also illustrates a longitudinal monitoring dashboard and feedback loop for treatment response tracking. This model is conceptual and requires prospective validation before real-time treatment modification can be recommended clinically.

Machine learning-driven treatment selection algorithms are being developed to integrate clinical parameters, biomarker profiles, and imaging data for therapeutic decision support. In principle, such systems could learn from treatment outcomes and improve prediction accuracy over time, but this capability remains largely conceptual in peri-implantitis. Real-time adaptation and dynamic treatment modification based on response monitoring require prospective validation before clinical use (146)(**Figure 4**). To synthesize these emerging modalities, **Table 1** provides a conceptual mapping of immunopharmacological targets, candidate delivery vehicles, and biomarkers that may be useful for future longitudinal monitoring.

**Table 1:** Conceptual mapping of molecular targets, candidate delivery vehicles, and biomarkers in peri-implantitis management.

Molecular target/mechanism	Precision delivery vehicle/strategy	Candidate biomarker or monitoring parameter
TNF-α/IL-1β	MOF-encapsulated antagonists (e.g., ZIF-8)	Cytokine levels in Peri-implant Crevicular Fluid (PICF)
RANKL-RANK axis	Sustained-release hydrogels (e.g., OPG-loaded)	CTX, TRAP5b, radiographic bone loss metrics
CXCL13+/CXCR2+ interaction (hypothesis-generating)	Experimental local delivery of chemokine-modulating agents	CXCL13 gradients in PICF (candidate marker)
M1 to M2 polarization	Mesenchymal stem cell-derived exosomes	CD86/CD206 ratio mapping
Resolution pathways (SPMs)	pH/ROS-responsive smart hydrogels (RvD1/RvE1)	Efferocytosis markers, Resolvin lipid profiles
Trained immunity/epigenetics	Nanocarriers delivering metabolic/HDAC inhibitors	Histone acetylation profiles in biopsied tissues
Tissue breakdown	Dissolvable multi-stage microneedles	aMMP-8 (Point-of-care Chairside Tests)

## 7 Clinical translation and future perspectives

### 7.1 Regulatory considerations and clinical trial design

Translation of advanced immunopharmacological approaches requires navigation of complex regulatory pathways. Combination therapies present particular challenges due to requirements for individual component validation and interaction studies (147). While currently limited in dentistry, parallels drawn from other fields like inflammatory bowel disease show that adaptive trial designs enable efficient evaluation of personalized treatment strategies (148).

Similarly, lessons from oncology highlight that companion diagnostic development must parallel therapeutic advancement to ensure appropriate patient selection (149). Biomarker validation requires large-scale clinical studies demonstrating clinical utility beyond analytical validity. Real-world evidence generation through registry studies provides additional safety and efficacy data (150).

### 7.2 Cost-effectiveness and healthcare economics

Economic analyses of precision medicine approaches must consider both development costs and potential long-term healthcare savings. Prevention of implant failure through early intervention may justify higher initial treatment costs, but this assumption requires dental-specific modeling (151). While specific economic models for AI in peri-implantitis are limited, studies in other medical fields, such as cardiovascular disease and oncology screening, suggest that early biomarker-guided intervention can yield favorable cost-effectiveness (QALY) ratios and support informed patient choices through shared decision-making tools. Future studies should adapt and test these economic models in dental implantology.

From a standard clinical perspective, the transition to real-time biomarker monitoring and AI diagnostics must justify its higher initial costs compared to traditional, low-cost periodontal probing and periapical radiographs. When factoring in the substantial cumulative costs of implant failure, explantation surgeries, and complex ridge augmentation, early diagnostics may prove economically viable in selected high-risk populations (152). However, whether AI-driven predictive prevention can substantially reduce recurrence rates in peri-implantitis remains an open question that requires prospective health economic modeling and clinical outcome data.

### 7.3 Technological integration and clinical workflow

Clinical implementation would require practical integration with existing dental practice workflows. Point-of-care diagnostic systems should provide rapid results without disrupting patient care (153). As demonstrated in cardiovascular risk prediction models (154), electronic health record integration can support comprehensive data collection for algorithm refinement; however, adoption in dental workflows should proceed with attention to validation, interoperability, and clinician oversight.

Training programs for dental professionals should address both technical aspects of new technologies and the interpretation of complex diagnostic results. Continuing education initiatives may support appropriate technology adoption and improve the likelihood of patient benefit, but outcome improvements should be evaluated empirically (155).

### 7.4 Future research directions

Multi-omics integration represents an important future direction in peri-implantitis research. Combining genomics, proteomics, and metabolomics data may help create more comprehensive patient profiles and generate hypotheses for personalized treatment. Single-cell technologies continue evolving and may provide greater resolution of disease mechanisms (156).

Real-time monitoring technologies utilizing smart implant sensors may eventually enable continuous assessment of inflammatory status. Wearable devices for non-invasive inflammation monitoring could broaden access to precision medicine approaches if analytical validity, patient acceptability, and clinical utility are established. Internet of Things (IoT) integration could support clinician-supervised treatment adjustments based on objective biomarker feedback, but automated decision-making remains a future possibility rather than a current standard (157).

Immunotherapy strategies may eventually draw inspiration from advanced oncology treatments, such as adaptations of CAR-T cell concepts (158) or ferroptosis modulation (159), though substantial research is required before these concepts can be adapted for local oral application. Combination approaches integrating multiple therapeutic modalities may prove beneficial, but superiority over single-agent therapies remains to be demonstrated.

## 8 Conclusions and clinical implications

The convergence of advanced molecular biology, artificial intelligence, and nanotechnology has created important opportunities for peri-implantitis research and future treatment development. Evidence from 2020–2025 supports immunopharmacological modulation as a promising next-generation strategy, but current data are not yet sufficient to establish it as the clinical cornerstone of management. Rather, these approaches represent a potential complement to mechanical debridement and conventional care, guided by molecular diagnostics and machine learning algorithms that still require validation.

The identification of immune signatures through single-cell transcriptomics, including the proposed CXCL13+ fibroblast-CXCR2+ neutrophil interaction axis, provides candidate mechanisms for further study in peri-implantitis. These discoveries, combined with advanced drug delivery platforms and regenerative medicine approaches, create opportunities to test strategies that may control inflammation while promoting tissue repair.

Clinical translation of these innovations requires continued interdisciplinary collaboration between immunologists, materials scientists, clinicians, and regulatory agencies. If validated, integration of biomarker-guided diagnosis, AI-supported risk stratification, and targeted immunomodulatory interventions may help clinicians move from reactive treatment toward more proactive prevention based on individual patient profiles.

The success of precision immunopharmacological approaches should ultimately be measured not merely by improved surrogate clinical parameters but by their ability to preserve implant function, enhance patient quality of life, and reduce healthcare costs through prevention of implant failure. Whether this field will produce a durable transformation in peri-implant disease management depends on rigorous mechanistic validation, well-designed clinical trials, and real-world effectiveness studies.

Future clinical practice may integrate multiple technological platforms in a coordinated approach: AI-supported risk assessment at implant placement, monitoring through validated biomarker or sensor systems, and personalized therapeutic interventions delivered through advanced biomaterial systems. This comprehensive precision medicine model remains an aspirational framework whose value must be demonstrated through rational, evidence-based immune system modulation and long-term clinical outcomes.
